# Challenges to competitive manufacturing in high-cost environments: checklist and insights from Swedish manufacturing firms

**DOI:** 10.1007/s12063-021-00193-0

**Published:** 2021-05-17

**Authors:** Nina Edh Mirzaei, Per Hilletofth, Rudrajeet Pal

**Affiliations:** 1 Department of Supply Chain and Operations Management, School of Engineering, Jönköping University, P.O. Box 1026, 551 11 Jönköping, Sweden; 2 Department of Industrial Product Development, Production and Design, School of Engineering, Jönköping University, P.O. Box 1026, 551 11 Jönköping, Sweden; 3 Department of Industrial Engineering and Management, University of Gävle, 801 76 Gävle, Sweden; 4 Department of Business Administration and Textile Management, Swedish School of Textiles, University of Borås, Allégatan 1, 501 90 Borås, Sweden

**Keywords:** Manufacturing location, Competitive manufacturing, Capabilities, Success factors, Challenges, Manufacturing industry

## Abstract

Research on competitive manufacturing (CM) in high-cost environments has earlier indicated that firms struggle to remain competitive and that manufacturing operations often have been offshored to low-cost environments. The purpose of this research is to explore and create a compounded view of challenges related to both internal and external environments of firms when operating in high-cost environments. This issue has been investigated through a qualitative case study involving five manufacturing firms in Sweden. This research has empirically derived the challenges associated with sustaining CM in high-cost environments and developed a prescriptive checklist. Seven main categories of challenges have been identified, ranging from a micro level related to product characteristics and employee involvement, to a macro level related to supply chain collaborations and industry systems. This research contributes to the existing literature on CM in high-cost locations by explaining and detailing what constitutes challenges in this kind of environment.

## Introduction

In striving for cost reduction, for several decades, manufacturing companies have shown a trend towards offshoring/outsourcing their operations to low-cost countries (Jensen and Pedersen 2012). However, the trend has recently started to reverse; reshoring to high-cost countries has gained momentum in several industries, owing to challenges related to the true total cost of offshoring, in terms of increasing logistics costs and difficulties related to managing offshore suppliers (Abbott 2007; Stanczyk et al. 2017; Eriksson et al. 2018). Although extant research has identified numerous motivations for repatriating activities from offshore locations to high-cost countries, our standpoint in this paper is that it is necessary to extend the focus beyond relocation to competitiveness in high-cost contexts. Tate et al. (2014) highlight that when companies start considering the “total cost of ownership,” “domestic manufacturing frequently appears to be a more total cost-effective choice” (p. 386). Motivated by the demand for the profitability of manufacturing in a high-cost environment, given that governments and shareholders are most likely unwilling to make up for the loss, there is considerable discussion on how to restore manufacturing competitiveness (de Treville et al. 2017). Recent literature has underlined the role of a vast array of operations and supply chain capabilities related to competitive manufacturing (CM) in high-cost environments (Ketokivi et al. 2017; Engström et al. 2018a), such as higher responsiveness (Moradlou et al. 2017), manufacturing flexibility (Martínez-Mora and Merino 2014; Johansson and Olhager 2018), greater control (Robinson and Hsieh 2016; Lampón and González-Benito 2020), long-term supply relations and higher demand for supply chain sustainability (Fratocchi and Di Stefano 2019).

Although such a capability perspective is extensively discussed in the operations management literature to assess the link between competitive manufacturing (CM), or manufacturing competitiveness and competitive advantage (e.g., Singh et al. 2007; Baines et al. 2009), the notion of CM is further broadened by its contextualisation in the operating environment where it is studied. Here, the notion of CM, drawn from the definition of de Treville et al. (2017), is referred to as the organisation of production activities and their industrial ecosystems in any given market environment such that it becomes a source of competitive advantage for the firm, classified in terms of Porter’s (1980) strategies of cost leadership, differentiation or focus. The specificities of the market environment determine the strategic fit with the manufacturing strategy (Lengnick-Hall and Beck 2005; Soosay et al. 2016). For instance, in high-cost environments typified by their cost structure, protectionism and dynamic market requirements (de Treville et al. 2017), CM is largely influenced by more specific capabilities to produce customised products using automation (Vinnova 2015; Pal et al. 2018), small-series responsive networks (Westkämper 2013; Yin et al. 2017) and high levels of labour market mobility (Stentoft et al. 2016a). Another prominent stream of research on high-value manufacturing (HVM) has offered a conceptual lens for organising manufacturing competitively in high-cost environments. However, largely evolving from value frameworks (e.g., Bowman and Ambrosini 2000), the HVM literature is more suited for pursuing the integration of services and offering a high-value proposition through the disciplines of product/service innovation, establishing process excellence, achieving higher customer value (Martinez and Bititci 2001; Huaccho Huatucoa et al. 2019).

Given the background of gauging the attractiveness of manufacturing in high-cost environments and the required competitiveness as means of spurring production relocation from low-cost to high-cost environments, the lens of HVM is beyond this paper’s scope; instead, a complementary perspective is demanded, as offered by CM. In this paper, CM guides the understanding of how firms’ critical operation capabilities influence their relocation from low-cost to high-cost environments, derived from internal factors and organisational resources (Alsmadi et al. 2011; McIvor 2013; Di Mauro et al. 2018; Lampón and González-Benito 2020). The better the fit, coherence and alignment among a firm’s resources that support these decisions, the better its performance will be (Sarmiento et al. 2008). This is primarily based on the creation of internal capabilities through the deployment of key resources that exist *within the organisation* (Soosay et al. 2016), and together with the ability to anticipate market trends and the operating environment, as well as to respond readily to changing customer needs (Stalk et al. 1992; Ancarani et al. 2019), it generates competitive advantage for firms (Sansone et al. 2017). Such a complementary perspective characterises the firms’ degree of manufacturing readiness to handle the eventual outcomes of their relocation decisions (Nujen et al. 2019).

Given these specific requirements determining CM readiness in high-cost environments, a detailed understanding of the challenges faced by firms that have relocated/reshored, or are planning to do so, is vital. Although the extant research on CM in high-cost environments has received increased attention since the 2017 special issue of the *Journal of Operations Management*, so far, we could not find studies that particularly explore and emphasise different challenges specifically faced by firms in such high-cost environments. Therefore, in this paper, we aim to explore and create a compounded view of challenges related to both internal and external environments of firms when operating in high-cost environments. Chosen as the context of this study, Sweden qualifies as a high-cost environment due to its high price parity index, purchasing power and wage cost (Green and Roos 2012). Based on the findings (Sansone et al. 2017; Pal et al. 2018) that the role of CM readiness in a high-cost context can be studied at various levels, we conceptualise that these challenges can be organised into *both* external opportunities related to markets and environments *and* internal resource-based (product, production, organisation) positioning, as well as into both operational and strategic levels (McIvor 2013).

This paper is structured as follows. First, the existing manufacturing competitiveness literature is scrutinised to identify the key challenges. Given the paucity of identifiable challenges directly related to CM in high-cost environments, key categories of challenges are identified and later used to conduct exploratory, semi-structured interviews with representatives of five Swedish manufacturing companies. An in-depth analysis of these challenges prescribes a comprehensive checklist of CM challenges specific to high-cost environments, which is beneficial for practitioners.

## Challenges to CM in high-cost environments

The notion of CM is of key significance and is applied to various levels, that is, country (e.g., labour laws), supplier network (e.g., contractual agreements), company (e.g., production capacities) and organisational (e.g., functions and top management teams), that largely determine the success of relocation (Van den Bossche et al. 2014; Wan et al. 2019). Van den Bossche et al. (2014) highlight a number of CM capabilities, related to availability of skilled labour, a firm’s internal capacity and assets, existence of processes and infrastructure to transfer knowledge and expertise, and internal management capability, which determine CM readiness and are also particular areas of concern or challenges if unmet. Many of these concerns about and challenges to high-cost CM are also consequences of long-term outsourcing, resulting in lack of innovation, unavailability of skills and knowhow, and non-existence of local supply networks (Martínez-Mora and Merino 2014; Engström et al. 2018b).

Regarding the purpose of this paper, the lack of CM readiness can be connected to many challenges specific to high-cost CM. For instance, at the product level, reduced innovation potential is a key challenge due to physical and often cultural distance. Autor et al. (2016) indicate the relative loss of innovation potential in manufacturing in high-cost environments due to the longstanding separation of manufacturing and product innovation in globalised manufacturing networks. It is estimated that 71% of US corporate patents that stem from manufacturing have suffered over the years due to reduced innovation, indicating that innovations and improvements may require direct involvement with the production process (World Trade Organization 2012, p. 76). Another major challenge faced by firms is related to the loss of operational capabilities along the production value chain. Wæhrens et al. (2012) highlight that the challenge facing the organisation of operations in firms in developed countries arises when “a company unlearns its internal operations capabilities and to too large a degree becomes dependent on external partners’ manufacturing capabilities”. In their study of Spanish footwear companies, Martínez-Mora and Merino (2014) find that the offshoring process has been so intense that some manufacturing stages and skills have almost disappeared from the home countries. Similar findings have been observed by Di Mauro et al. (2018) and Engström et al. (2018a). Among other process-level challenges, high labour cost in production has also been reported as a crucial issue (see Gray et al. 2013; Bailey and De Propris 2014; Martínez-Mora and Merino 2014). Kotabe et al. (2008, p. 84) highlight how “because of substantial labor cost differentials, the existing production location, which is normally the firm’s home country in the early stages of internationalization, is no longer seen as competitive”.

In this connection, organisational learning is a key aspect of developing innovation and competitive processes as needed in a changing market (Chaston et al. 2001). However, with several years of offshoring and outsourcing, as pointed out by Bailey and De Propris (2014) in the case of mature industries, such as the UK automotive industry, the lack of skilled labour and trained management has widened the skill gaps between home and host countries. In the labour-intensive industry (e.g., textile and apparel) context, Pal et al. (2018) and Sirilertsuwan et al. (2019) have highlighted the lack of skilled and knowledgeable labour as a key challenge to productivity increase, which requires large financial and time investments by firms in order to bridge the knowledge gap. However, such efforts and investments in process and skill development (by industry associations and local retailers) could be risky, time-consuming (Van den Bossche et al. 2014) and just an additional cost to be incurred by retailers without an adequate market. Furthermore, in contrast to reduced overhead and logistics-related costs, CM in high-cost countries can increase both direct and indirect costs. Pal et al. (2018) underline major issues for increasing costs in relation to customer order volume and mix fluctuations, resulting in higher overhead allocated per product due to low scale and high seasonality. At the strategy level, such loss of skillset and managerial capability demonstrates weakness in the decision-making process when it comes to systematic planning of the supply network that is much needed to access both local and foreign factor markets or control distribution channels in order to reach out to globally positioned customers (Fratocchi et al. 2016).

From the supply chain side, such challenges can be the results of either the disappearance of the home-country supplier network or the lack of the required commercial levels of materials. Regarding the first cause, Van den Bossche et al. (2014, p. 27) argue that “companies that return their own manufacturing operations may still have to rely on suppliers from overseas, at least until the economics for the supplier also drive them to return to the U.S.”. As for the second cause, Ashby (2016) highlights how growth in offshoring has resulted in the decline of the once strong wool industry in the UK, thus the loss of the breeds relevant to the production of high-quality wool by local suppliers. Such challenges in the decision-making process may also arise from the market side due to the local market’s low attractiveness (Gray et al. 2013; Bailey and De Propris 2014; Engström et al. 2018a). Firms that largely depend on domestic demand are “vulnerable to local economic conditions and high-level decisions” (Soosay et al. 2016, p. 17), that is, location decisions made by strategically important customers. In the study of Soosay et al. (2016), many of the case companies from Australia and Sweden highlight that the main CM challenges and constraints are related to the market-related demand uncertainty associated with high seasonality or managing large variations.

Other more country-level factors, specifically institutional and macro-economic ones, such as stricter labour market conditions and environmental dynamics, also pose direct challenges to CM if firms lack readiness in high-cost environments (Zhai et al. 2016; Nujen et al. 2019). For instance, Business Birmingham (2013) highlights how restrictive regulations, such as visa restrictions or commercial legislation, have resulted in higher costs in the UK. Any employee who is recruited as a result of reshoring needs to be automatically enrolled in a pension plan, resulting in higher costs related to employment regulations and pensions that pose an obstacle to the expansion of home manufacturing operations. As indicated by Zhai et al. (2016), another reason is related to ineffective government incentives that have not really facilitated CM in Europe compared with that in the US. From an environmental perspective, Pal et al. (2018) note that firms have to comply with stricter jurisdictions, regulations and laws when producing goods. For example, several studies have emphasised the increasing awareness about environmental issues in the green manufacturing context, which can then put competitive pressures on and restrictions to manufacturing in high-cost, developed countries (Ellram et al. 2013; Gray et al. 2013; Fratocchi and Di Stefano 2019; Orzes and Sarkis 2019). Based on a survey with manufacturing firms in the Midlands of the UK, Bailey and De Propris (2014) highlight that high energy and raw material costs are considered key challenges to UK manufacturing, in sharp contrast to the situation in the US, where repatriation has partly been encouraged by lower energy costs.

Table 1 summarises these major challenges to CM in high-cost environments (as found in the literature) from the within-organisation perspective and categorises them into seven levels (product/service, production, organisation, strategy, supply chain, market and general environment), based on the theoretical underpinnings cited above. The categorisation is based on the organisational logics provided by Ketokivi et al. (2017), among others, and allows the challenges to be associated with different entities at both the operational and the strategic levels. This categorisation further guides the exploratory interviews.

**Table 1:** Summary of challenges and their categorisation for manufacturing in high-cost environments

Challenges to CM in high-cost environments	Indicated in	Challenge categorisations used in this paper
Reduced innovation due to long separation of innovation and production process	World Trade Organization 2012; Autor et al. 2016; Stentoft et al. 2016b	Product/service related (P)
Inadequate production capacity (e.g., absence of manufacturing stages, resources and internal capabilities)	Wæhrens et al. 2012; Bailey and De Propris 2014; Martínez-Mora and Merino 2014; Fratocchi et al. 2016; Engström et al. 2018a; Nujen et al. 2019; Lampón and González-Benito 2020	Production process related (PP)
High labour cost (and cost differentials)	Kotabe et al. 2008; Gray et al. 2013; Bailey and De Propris 2014; Martínez-Mora and Merino 2014; Engström et al. 2018b; Eriksson et al. 2018	
Absence of skilled labour (e.g., shortage of qualified staff, lack of tacit knowledge and core competencies)	Bailey and De Propris 2014; Engström et al. 2018a; Pal et al. 2018; Sirilertsuwan et al. 2019	Organisation related (O)
Lack of finances and assets	Bailey and De Propris 2014; Van den Bossche et al. 2014; Engström et al. 2018b; Pal et al. 2018	
High cost (overhead)	Eriksson et al. 2018; Pal et al. 2018	
Lack of systematic planning of supply networks	Fratocchi et al. 2016	Strategy related (S)
Absence of local supplier (e.g., limited knowledge and less access to raw materials)	Bailey and De Propris 2014; Van den Bossche et al. 2014	Supply chain related (SC)
Lack of attractiveness to local consumers and local demand uncertainty	Gray et al. 2013; Bailey and De Propris 2014; Soosay et al. 2016	Market related (M)
Stricter labour regulation (e.g., lack of government incentives, lack of labour market flexibility)	Business Birmingham 2013; Stentoft et al. 2016a; Zhai et al. 2016; Engström et al. 2018a	General environment related (E)
Stricter environmental regulations (e.g., requirement for reduced carbon footprint, environmental standards and laws)	Pal et al. 2018; Fratocchi and Di Stefano 2019; Orzes and Sarkis 2019	
High energy costs	Bailey and De Propris 2014; Van den Bossche et al. 2014; Zhai et al. 2016	

## Research method

Owing to the under-researched and complex phenomena (i.e., challenges with CM in high-cost environments), we adopted an exploratory research design, involving five case companies (Meredith 1998; Voss et al. 2002; Yin 2009). The unit of analysis was defined at the plant level, from the within-organisation perspective, to understand what challenges were perceived by employees with different responsibilities and who were working at various organisational functions.

The five selected case companies were participating in the then-ongoing research project focusing on CM in high-cost environments. They were chosen based on (1) their local sites’ abilities to remain competitive on the global market, according to the criteria of their corporate group and depending on the profit margins in the industry; (2) their size, as it was believed that they should not be too small for the challenges related to several organisational functions to be identified; (3) their roles as part of global corporate groups with worldwide manufacturing, as this was believed to provide the interviewees with a deeper understanding of global conditions; and (4) their willingness to participate in this study. We focused on the local, Swedish manufacturing sites.

In this paper, the participating companies are referred to as FastCo, a manufacturer of fastening tools and fixing solutions for office and construction (the only company out of the five in this study that had initiated reshoring efforts); PlastCo, a manufacturer of plastic packaging; ComCo, a manufacturer of different types of solutions for communications technology; WashCo, a manufacturer of professional washing equipment; and AutoCo, a manufacturer of drivelines and interiors for the global automotive industry. Table 2 depicts the characteristics of the five case companies at the time of the study.

**Table 2:** Company characteristics at the time of the study

Parameters	FastCo	PlastCo	ComCo	WashCo	AutoCo
Global corporate group’s headquarters	Sweden	Sweden	Sweden	Sweden	Norway
Company size (number of employees on local site)	250	300	800	500	550
Annual turnover (MSEK)	600	1550	-	1700	900
Company ownership	Private	Private	Private	Private	Private
Customers	Consumer	Business	Business	Business	Business

The empirical data consist of 56 semi-structured open-ended interviews (Bryman and Bell 2015) with 51 employees. Lasting around one hour each, the interviews were conducted by several of the researchers involved in the research project. At least two researchers participated in each interview to ensure the study’s validity (Maxwell 2005). The interviewees were selected based on their positions and knowledge about the operational and the strategic aspects of their particular plant’s organisation and operations. This selection was made to capture as holistic a picture of the organisations’ challenges as possible, while obtaining many different views on what constitutes this holistic picture. The interviewees covered functions such as sourcing/purchasing, product development, production, sales/marketing, finance, logistics/supply chain and the CEO/plant manager position. However, not all functions were captured in all companies (Table 3). All interviews followed predefined interview guides, partly adjusted according to each interviewee’s role. The interview guide was structured into five sections to gain general knowledge about the case companies (i.e., company information and organisational structure), the personal backgrounds and experiences of the interviewees, and more specific challenges (i.e., characteristics of CM in Sweden, operations and supply chain strategies, and change management). The challenge-related questions were asked under the seven categories identified in the extant literature, with the specific challenges (per Table 1) constituting the guiding words. To ensure reliability, the interviews were audio-recorded and transcribed.

**Table 3:** Interviewees

Function	FastCo	PlastCo	ComCo	WashCo	AutoCo
Sourcing/ purchasing	1	1	1	-	1
Product development	1	-	1	1	1
Production	4	3	4	4	3
Sales/ marketing	1	2	-	-	1
Finance	-	-	-	3	1
Logistics/ supply chain	1	3	5	1	1
CEO/ Plant manager	1	1	1	1	2

The data analysis followed the three-phase procedure advised by Miles and Huberman (1994) – reduction, data display and conclusion/verifications – with support from the NVivo 11 software (QSR International). All interviewees’ transcriptions (i.e., the sources) were reviewed, and quotes related to challenges associated with CM in high-cost environments were derived, guided by the seven categories (Table 1). Based on the logic in NVivo, a multitude of codes were created. This initial stage of the data analysis was iterative, meaning that for every new interview transcript analysed, there was the potential of new codes and a new code structure to emerge. This identification of the specific challenges was (1) a result of the interviewees’ own descriptions and vocabulary, partly based on their organisational belonging and their definition of the challenges in the context closest to their daily work tasks, and (2) based on the categorisation of challenges in Table 1. Once the challenges identified empirically were categorised and described, they were analysed both from within each case and between cases. The identified challenges were compared with those derived from the existing literature. Similarities and differences between the challenges identified in Table 1 and the challenges in the empirical data helped us identify three key propositions for future research.

To validate the findings, the participating case companies were involved. The findings were presented to all five companies, and through discussions with company representatives, it became clear that (1) the identified challenges and the way they were clustered and categorised seemed to depict the perceived reality by the interviewees, and (2) the structuring of the challenges into a checklist was perceived as easy to comprehend throughout the organisations. The company representatives intuitively understood the logic and believed that they could work further with this structuring of challenges in their own organisations.

## Empirical findings

From the five case companies, we empirically derived the challenges associated with CM in high-cost environments under the seven categories presented in Table 1. Each of the seven main categories of challenges has been found to include several sub-challenges that specify in detail the characteristics of the perceived challenges. Appendix 1 details these sub-challenges in each case. Similar sub-challenges may appear under several categories, depending on the perspective taken – operational or strategic, internal or external. For example, lean appears *both* as methods and techniques at the operational level related to the production process *and* as a philosophy at the strategic level.

### Individual case analysis

In this section, we focus on the specific situations of each of the five case companies – what their greatest challenges are and what is especially important for them to address in order to remain competitive.

FastCo identifies the most challenges. It faces substantial market changes, with a decreased interest in their products. It is therefore not surprising that FastCo associates most of its challenges with the need to improve its production processes. The company faces customer demands for lower volumes, calling for higher levels of flexibility, at the same time as it identifies the need for increased automation along with the reduction of the number of person-hours per product. The production needs to be rationalised, with better internal delivery service. Organisation-wise, the demands for changes in the production processes imply the need to focus its employees’ efforts on continuous improvements, requiring involvement by highly skilled workers and intra-organisational collaborations. Such initiatives are difficult for the company to accomplish as the employees perceive a lack of strategic direction and long-term focus, limited by the perceived lack of interest by the owner in the local plant.

PlastCo identifies the least number of challenges among the five companies. Furthermore, the nature of the challenges that it pinpoints differs from those of the other companies. The challenges of this company are mostly associated with identifying the right skills and knowledge for running its complex production processes. The needs to involve the employees in the decision-making process and to utilise the knowledge within the organisation are emphasised. However, related to the political factors, the company witnesses considerable difficulties in finding the few individuals available in the labour market with the right competencies and in attracting them to the company’s rural location. The company also addresses challenges related to political decisions at the European Union level, leading to what is perceived as unfair market conditions for producers in high-cost countries.

ComCo’s challenges primarily relate to organisational and strategic aspects, where intra-organisational collaboration is emphasised as a key challenge to address in order to remain competitive. The interviewees perceive both the local plant and the intra-group relations as difficult to oversee with too many silos and sub-optimisations due to the lack of a coherent strategy, which is operationalised throughout the organisation. These tendencies can also be regarded as related to the entire supply chain, where the company is challenged by discrepancies and the lack of understanding of the customers’ needs, functioning more as individual companies than as a coherent supply chain.

WashCo encounters more challenges related to its products than the other four companies do. The company faces declining market demands and therefore identifies the need to provide the market with innovative, premium products, easily ordered and with rapid new product development processes as crucial for its survival. Such changes call for clear strategic plans and committed owners. However, the company is constantly challenged by the listing on the stock exchange and the rapid demand for a steady cash flow. Regarding the politically related challenges, the company is worried about not being able to attract the right candidates from the workforce, as well as the educational system’s inability to equip students with the required competencies. WashCo also faces challenges related to legislation and regulations of its market.

AutoCo’s greatest challenges are associated with its supply chains and the location’s proximity to the customers as the automotive industry is extremely dependent on just-in-time deliveries and does not have room for production stoppages in order to remain competitive. Due to this relationship with its customers, AutoCo is also challenged by its own suppliers and their uncertain abilities to not only provide components according to the agreement but also to view the supply chain as a value chain where each part needs to focus on cost reduction and improvement efforts to make the production as efficient as possible. The local plant is also challenged by competition from within the group, top-down management from the group and internal problems with organisational boundaries among its various functions at the same time as the market requires increasingly shorter new product development processes.

### Comparative case analysis

Appendix 1 provides supporting evidence from the empirical data for each sub-challenge, as observed across the case companies. The table in Appendix 1 shows that not all challenges are relevant for all five companies since they represent different industries and therefore face slightly different challenges. However, the way that the challenges are expressed indicates a pattern that allows a cross-case analysis, aimed at identifying and understanding the deeper, comparative picture of challenges for companies in high-cost environments. The empirical data presented in Appendix 1 reveal some patterns of relevance for understanding what challenges these five companies encounter at a group level, allowing us to obtain some ideas of what may constitute challenges in high-cost environments. In the following section, we elaborate on those patterns based on the categorisation provided in Table 1. A detailed mapping of the challenges across the case companies is also provided in the form of a checklist in Table 4.

**Table 4:**
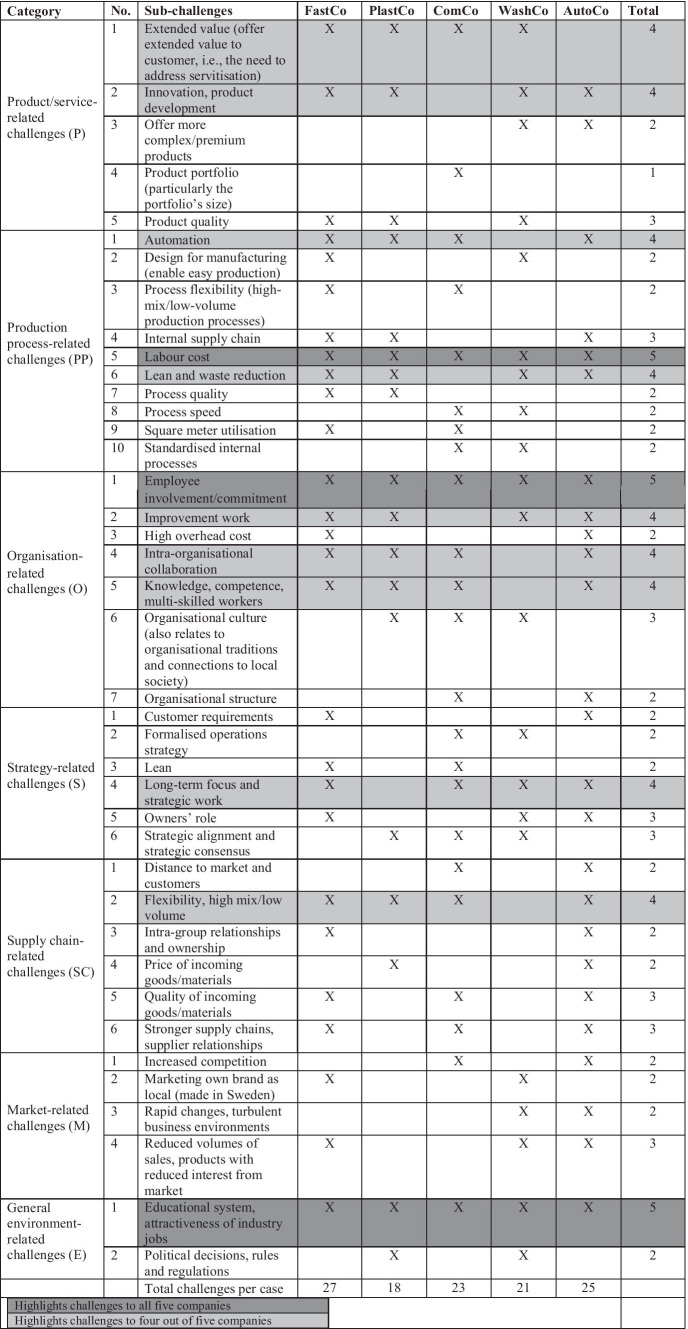
List of challenges

#### Identifying similarities in each challenge category

The product-related challenges concern a company’s ability to provide an extended value of the product for the customer. This incorporates challenges related to the customer offer and the need to address servitisation and the offering of total solutions. Furthermore, these sub-challenges involve the innovation and product development aspects and the need to offer more complex, value-adding products in a wide portfolio where the quality is consistently high for the whole product range. These sub-challenges are primarily viewed as possible to address internally but are heavily impacted by changes to the external environment as they are closely related to the (changing) customer needs.

The production process-related challenges brought up by the interviewees concerned the level of and technology for automation, as well as its financial underpinnings. Related to this, the concept of design for manufacturing and the need to enable production that is as easy as possible are addressed by two of the companies. Challenges associated with process flexibility (the need for high-mix/low-volume production processes), the internal supply chain, lean production and waste reduction, process quality, process speed, square meter utilisation in the production facilities and standardisation of internal processes are also addressed. The most prominent sub-challenge in this category that is addressed by all five companies relates to the labour cost and the need to improve the utilisation of person-hours per produced product/unit/item. The identified sub-challenges imply that in each company’s internal production processes, there is the need to address the traditional competitive priorities for manufacturing, such as quality, speed and flexibility, by means of implementation of technologies (e.g., automation and philosophies, such as lean production).

The organisation-related challenges (i.e., challenges on the local site from an intra-organisational perspective) primarily refer to how the work and the different functions on the site are organised. These sub-challenges concern employee involvement and commitment, improvement work, high overhead cost, intra-organisational collaboration, knowledge, competence, multi-skilled workers, organisational culture related to organisational traditions and connections to the local society and organisational structure. As shown in Appendix 1, many of these sub-challenges concern *soft* issues, such as the need for skilled employees who are involved in improvement work. This work is primarily related to their own contextual level, as well as to the overall organisational level. Furthermore, there is the need for employees who can work in complex environments where flexibility and collaboration skills are essential.

The strategy-related challenges concern customer requirements, a formalised operations strategy, lean implementation and work, the organisation’s long-term focus and strategic work, the role of owners and the call for strategic alignment and strategic consensus within and among functions. These challenges capture the internal perspective of strategic work and further emphasise challenges associated with individuals’ abilities to work together and understand their own roles in the company, as well as the company’s role in its corporate group and in relation to its competitors.

The supply chain-related challenges concern both organisational internal and external aspects and include the geographical distance to the market and customers, the flexibility and the organisation’s ability to keep a high product mix at low volumes. They also concern intra-group relationships and ownership, the price and quality of incoming goods and materials and the need for stronger supply chains, focusing on supplier relationships. Hence, these sub-challenges are directly associated with the local site (e.g., its location), while taking a more external viewpoint and focusing on challenges related to relationships both upstream and downstream in the supply chain, including within the firm’s own corporate group.

The last two categories of challenges are to some extent intertwined, as market conditions are often influenced by political decisions. However, they are here separated as their origins differ to some extent. The market-related challenges depend on market-based factors, such as customer demands and competitors’ actions, and often indicate substantial changes at a global level. The market-related challenges identified here concern increased competition, the ability to market each company’s own brand as local (“made in Sweden”), rapid changes, turbulent business environments and reduced volumes of sales and products with declining interest from the market. Meanwhile, the politically related challenges seem to stem from other macro-economic factors, such as those associated with the educational system and various legislations. In this research, the politically related challenges concern the form and function of the national educational system, the attractiveness of jobs in industries at the national level and the impact of political (governmental) decisions, rules and regulations on the ability to operate a profitable business in Sweden.

#### Highlighting the most crucial challenges

When analysing the empirical data, some challenge categories seem to be of greater importance than others for the studied companies’ possibilities to operate competitive production sites in the Swedish high-cost environment. There are many identified challenges related to the production process, as indicated in Appendix 1. Although in general, the production process-related challenges do not indicate the criticality of the companies’ competitiveness, one of its sub-challenges does. Labour cost is identified as a challenge by all five companies. PlastCo emphasises the total labour cost as a challenge. For AutoCo, this is an issue for Swedish production in general and must be balanced with automation. Meanwhile, FastCo, ComCo and WashCo focus more on the direct labour cost, linked with the production efficiency and the number of person-hours used per product. Notably in this regard, the need for automated manufacturing is also emphasised by four out of the five companies. Two aspects of production system development are clearly interlinked and often directly associated with each other. Despite this focus, it seems that the companies do not identify them as the ultimate driving forces for location decisions. Rather, the organisation-related and politically related challenges are apparently of greater importance. In other words, the challenges with a high-cost environment localisation cannot primarily be linked to issues related to technical aspects of production but to aspects at a more aggregated level. Concerning the organisation-related challenges, all five companies acknowledge the importance of employee involvement/commitment, and four of them emphasise improvement work, intra-organisational collaboration and knowledge, competence and multi-skilled workers as challenges that should be addressed to remain competitive. This focus on an organisational internal level indicates the companies’ potential for improvement in addressing these challenges without being dependent on external factors. The opposite is the case for the politically related challenges. The companies’ statements bear witness to a certain frustration regarding the educational system and how little the individual companies can do about the external factors associated with it. In addition to the concerns regarding the national educational system and its focus on jobs in industry, or lack thereof, the companies also address European Union regulations and the attractiveness of industry jobs at a general level as factors challenging their survival. 

While previous research to a large extent has had a cost-based and to some extent, a macro-economic focus, our empirical findings offer a within-organisation perspective, drawing on individuals’ perceptions of challenges to their work environment at a plant level, paying attention to operations and supply chain management and capturing the micro-level challenges in high-cost environments.

## Concluding discussion

Given the purpose of this research (i.e., exploration of the challenges), we identify seven main categories of challenges related to both internal and external environments of firms when operating in high-cost environments. These challenges range from a micro level, related to product characteristics and employee involvement, to a macro level, dealing with supply chain collaborations and industry systems, thus contributing to synthesising a checklist (Table 4). The comparative case analysis using this checklist indicates that the sub-challenges related to labour costs (PP5), lack of employee involvement/commitment (O1), and the educational system’s inadequacy in equipping students with the required competencies and low attractiveness of industry jobs (E1) are identified as the most crucial impediments to remaining competitive. Nine additional sub-challenges are prioritised by four of the five companies. Based on the comparative mapping of the challenges in Table 1, and the companies’ reasoning summarised in Appendix 1, our study contributes to capturing a structured and more compounded view of the challenges when dealing with multiple types of challenges together. These compounded challenges are captured below in the form of three propositions.

First, in line with several other studies (e.g., Martinez and Bititci 2001; Huaccho Huatucoa et al. 2019), our research resonates with the importance of creating extended customer value yet acknowledges this as a crucial challenge, demanding overall improvement of customised offerings (e.g., through end-to-end solutions, improved logistics, better promotion (P1)). While earlier research (e.g., Singh et al. 2007; Soosay et al. 2016) has emphasised the importance of automation for generating such customised value offerings through CM in high-cost environments, our findings additionally highlight several related key challenges, for instance, in terms of finding the right level for producing small batches, the need for continuous investment, attaining break-even financial returns (PP1), as well as keeping high levels of product innovation through rapid technological developments (P1, P2). In such an agile climate, it is invariably a challenge to maintain higher process efficiency through lean improvements in order to maintain the product price within limits, as indicated in PP6 and O2. At the supply chain level, this further increases the demand for smaller batches, higher flexibility and quicker reactions from proximity suppliers (SC2). Hence, we formulate our first proposition.

### *Proposition 1:*

*The demand for flexible production of small batches in high-cost environments is crucial for delivering higher customer value; however, this increases product, process and supply chain costs related to technology and efficiency improvements.*

Second, while increased automation could potentially reduce reliance on high labour costs, which poses a huge challenge to organising CM (PP5), it would also require higher innovation and the appropriate skill set to handle technology. This demands an accelerated transition to more knowledge-based manufacturing to handle automatic processes, multiple tasks, and so on. Our study’s results complement earlier findings (e.g., Pal et al. 2018; Nujen et al. 2019) related to the importance of skilled labour regarding both technical expertise and management skills and acknowledge the challenges associated with the shortage of such knowledge base (E2, O5). Additionally, the empirical data have revealed the significance of decreasing labour costs, while balancing the investments in automated equipment. This somewhat creates a paradoxical situation since reducing labour intensity necessitates greater reliance on knowledge intensity and automation, which in turn requires increased initial investments in training and education, skill development and technological innovations. As pointed out by Jensen and Pedersen (2012), there is a global quest for a knowledgeable workforce, and as illustrated by the empirical data, there are challenges involved with high-cost environments not providing the right types of skills and education to meet the market requirements (E2). Thus, a second proposition has been formulated.

### *Proposition 2: *

*Re-establishing CM in high-cost environments requires cost considerations in terms of balancing knowledge intensity and labour intensity.*

Finally, our study also illustrates the need for higher internal integration through closer collaboration among individuals, functions and departments. The challenge that arises is referred to as silos resulting in sub-optimisation (O4) and is emphasised as problematic in creating an end-to-end perspective, implying a holistic viewpoint. In line with the findings of Ketokivi et al. (2017, p. 27), such silos generate a higher degree of decoupling in organisational process structures, illustrated by the case companies as gaps between product development and production and between production and logistics, thus hindering any organisational change. The silo mentality also results in less communication between departments and individuals, thus reducing employee commitment (O1). Thus, the current improvement works in the case companies largely have a short-term focus (i.e., productivity-oriented), while there is a lack of strategic direction and long-term focus on enhancing value offerings, return on investment and employee commitment (S4). We conclude with our third proposition.

### *Proposition 3: *

*The current short-term, productivity-oriented focus when organising CM in high-cost environments collides with the need for developing a long-term vision for sustainable growth.*

### Implications for theory and practice

In relation to previous studies (e.g., Ellram et al. 2013; Tate et al. 2014; Soosay et al. 2016), the prescriptive checklist developed in this paper provides additional knowledge and insights into challenges associated with CM in high-cost environments. Previous research to a large extent has had a cost-based and to some extent, a macro-economic focus. In contrast, our research offers a within-organisation, contextual perspective, drawing on individuals’ perceptions of challenges to their work environment at the plant level and focusing on operations and supply chain management. In comparison to previous research, our study thus offers (1) better structuring of challenges as internal or external to the organisation, (2) an analysis on both operational and strategic levels and (3) support to a compounded view of challenges related to seven categories.

This study also provides valuable insights to operational managers, with a checklist of the challenges to be considered when organising and repatriating production to high-cost environments. The checklist may be used as a practical tool to verify that important aspects are considered at various levels simultaneously. The overview provided by the checklist also enables cross-functional understanding and visualises the complexities involved in remaining competitive.

From a managerial perspective as well, it is crucial to ensure that all employees involved in decision-making processes work and coordinate among themselves with a holistic perspective towards the challenges on both operational and strategic levels, beyond silo thinking. In this context, the prescriptive checklist can be used as a tool to communicate the importance of different aspects (challenges and opportunities) and integrate different decision-making groups and departments in the organisation.

### Limitations and scope for future research

Given that the CM challenge checklist is derived from a case study on five Swedish manufacturing companies, the results are specific to a particular high-cost region; thus, the findings’ generalisability can be considered somewhat limited. As our study is explorative in nature, there is a need for future research to gather empirical data from similar and other research settings in high-cost environments to reinforce the validity of our findings. This would require using the prescribed checklist as a deductive model to conduct extensive survey-based work.

Our in-depth case research reveals several similarities with the challenges identified in earlier studies, along with a number of unique challenges. While this exploration of challenges in high-cost environments offers both structured and compounded views on what these challenges constitute, further research is needed to explore their reasons and effects. Moreover, there is a need for research to provide practitioners with potential prescriptive solutions for managing these challenges.

Meanwhile, Ellram et al. (2013) supplement these challenges with those related to country risk and supply chain interruption, such as natural disasters, environmental issues, terrorism and political instability. These types of challenges do not arise in this study, mainly due to the non-appearance of such macro-level challenges, owing to the higher political stability in most European nations, perhaps the Nordic countries in particular. However, with the currently impending higher market volatility (e.g., due to Brexit or the COVID-19 pandemic), such external risk-driven challenges could be added to the checklist in the future.

## Appendix 1 Within case analysis of challenges and identification of emerging patterns

**Table:** 

Category	Sub-challenges	FastCo	PlastCo	ComCo	WashCo	AutoCo
Product/service-related challenges (P)	Extended value	Needs to improve promotion of several items together to satisfy the customer’s need of complementary products in one package with the main product	Needs to market the product as “the value of less cost per used product”, rather than simply a product for wrapping	Needs to offer a total solution (smart, value adding) simple for the customer to handle. Further, need to offer an “end-to-end” solution, including easier ordering process and packaging of product that enables rapid start up for the customer	Needs to improve logistics: “there are great opportunities in e-commerce and we need to offer the customer a track and trace service”	
	Innovation, product development	Facing declining sales volumes due to changes in the market demands. Need to “find the next big thing” and carefully follow market changes while aiming for new directions and a higher level of electronics in the products	Challenged by competitors who easily can copy the product. Need rapid innovation to always serve the market with new products		High level of innovation and rapid new product development of advanced products are key	Needs to keep high level of innovation and rapid new product development of advanced products. Faces challenging technological shifts in the market segment
	Offer more complex/premium products				Faces risks of declining appeal for their “premium” products, ending up in a situation competing on price	Needs to find “a situation where more complex products really benefit from high levels of automation”
	Product portfolio			Faces large costs for keeping a wide range of products; material, production facilities, system maintenance		Needs to widen the product portfolio to reduce the dependency on a few customers
	Product quality	High quality is a prerequisite. Challenged by discrepancy in customer perception; “good quality” is to always deliver what the customer wants	Product is expensive and easily imitable; quality focus has deteriorated. Challenged by competitors catching up on high quality		Offers expensive, premium, product and must “offer really good products”; targeting zero defects	
Production process-related challenges (PP)	Automation	Needs to find right level of automation for small batch production by balancing automation and high investment costs. Needs to find “simple automation solutions, semi-automate without too large investments” and “flexible, smart, simple, automation”	Process industry calls for continuous investments in new equipment	Needs automation with the latest technology, constantly updated		Needs to define “automation break-even point” to avoid unnecessary investments, i.e., the point where high levels of automation only are beneficial if they are handling high volume production
	Design for Manufacturing	Essential to focus on “products that are easier to manufacture, and thereby cheaper			Needs to develop new products that are easy to produce, “too complex production may result in quality issues”	
	Process flexibility	Faces customer demands for fast, low volume deliveries and needs to “be better at producing low volumes with short, flexible set ups		Necessary with flexible processes to address demand for “shorter and shorter delivery lead times”		
	Internal supply chain	Needs to be “better at the internal delivery service, especially from production to warehouse”	Needs to improv internal processes and internal delivery precision “to 95 percent to become more efficient”			Needs to keep better track on the material in-house
	Labour cost	Needs to decrease the amount of man hours per product, as “we cannot compete on labour cost, so we need to work more efficiently” and “we need to reduce waste in the production process to keep the labour cost down”	Faces challenges associated with labour costs, “including benefits such vacation”	Needs to “utilise our person-hours and minimise all waste to assure efficiency and thereby keep the labour cost at a minimum”	The direct labour cost is “a very important cost to keep low”	“Labour cost is a large drawback for Swedish production, need to balance it with high automation levels”
	Lean and waste reduction	Needs to reduce waste throughout production	Produces too much scrap material		Seems to have lost focus for why lean is important and needs to “map the waste to the processes to be more efficient”	Needs to constantly reduce waste and identify value adding activities
	Process quality	Challenged by different views on quality: as “the foundation of all other work” or as something more relevant in the past; “it has had to stand back to the focus on speed”	Needs to introduce new quality controls to “reduce the customer complaints”			
	Process speed			Needs to handle incoming orders faster through simple fast internal flows	Faces demand for shorter and shorter delivery lead times	
	Square meter utilisation	Needs to free up space to rationalise production		Consolidates space to save money and increase profitability		
	Standardised internal processes			Needs to standardise internal processes to identify synergy effects and get a shared understanding throughout the company	Needs to “focus greatly on bringing in a better standardised work, too many routines fail a bit too often”	
Organisation-related challenges (O)	Employee involvement/commitment	Faces difficulties getting “everyone on board” during organisational change, concerning communication and in assuring that improvements are initiated for competitiveness, not for headcount decrease or offshoring	Needs to “focus more on the employees and make them feel involved and engaged in what we do”, emphasising: “every individual is important, and we need to utilise their competence”	Needs “motivated, committed employees” with “willpower and passion for doing a great job”; “we need all hands and feet and brains, really”. Problematises getting everyone on board: “to be aware of the own role in the operations”	Challenging to “involve all employees, not to depend on a few driving spirits to possess the knowledge and the will to improve”	Strives to involve everyone in identifying areas of improvement and to give them the possibility to develop within their own roles
	Improvement work	Needs to “find improvements constantly to assure that our product price does not go too high”. Believes in “evolution instead of revolution”, stressing both the importance of dissatisfaction with the current state, a strive to do better and a positive attitude towards changes	Needs skilled operators able to identify problems and an organisation open for problem solving through interaction		Needs to constantly improve to keep up with the competitive situation in the market	Struggles to define documentation structure capturing improvement activities and to find time for improvement work in a slim organisation
	High overhead cost	Needs to reduce overhead costs as cost for material is set on a global market and difficult to impact				Faces unnecessarily high overhead costs due to “a lot of support functions that might be better to outsource”, since supporting staff does not “generate income”
	Intra-organisational collaboration	Needs to consider production and modularisation already at the product development stage and increase collaboration among sourcing, product development and sales to assure that decisions are not sub-optimised	Needs to include workers in machine investment decisions	Needs “a much closer collaboration among functions to assure understanding of cause and effect”. Currently organised in “independent silos” risking sub-optimisation. “The hierarchical structure is too slow to maneuver, we need more networking, more agile groups.” Further, needs to involve blue collar workers: “white collar workers cannot make decisions without involving the people who later are going to do the work”		Needs to remove some of the inherited organisational boundaries and improve collaborations. Needs to incorporate (automated) production process knowledge early in product development, assuring production efficiency. Needs more communication and interaction in interface between product development and production. Problematises recent relocation of sourcing/purchasing to another city
	Knowledge, competence, multi-skilled workers	Needs multi-skilled workers, production requires craftsmanship and employee flexibility: “not enough to have machine competence, need to be more knowledgeable about whole value chain”. Further, faces need of production technicians and employees with knowledge on tools, machines and equipment maintenance	Needs to “educate younger people”. Facing too many retirements within ten years and needs to “ensure their knowledge stays in the organisation”. Further, increased levels of automation leads to “completely changed demands on work force: skilled set up technicians able to handle advanced automated equipment”	Needs wider range of competence, including comprehension of customer needs, and how to influence the product design. Worried by a too old workforce where there are no youngsters with right knowledge available		Faces challenges associated with finding the right competence for tool and die makers
	Organisational culture		Need to alter mindset regarding costs with everyone aware of the importance of profitability	Needs good leadership encouraging collaboration and challenge traditions. and	Faces a challenge where “the local culture is not as prevalent as it used to be. Employees have lost their sense of belonging to the company and its importance for the local community”	
	Organisational structure			The organisational structure challenges the organisational strength with its “too hierarchical organisation and decision processes”, risking separate silos with limited collaborations		Challenged by the corporate group’s top-down management style, mirrored in the organisation at the local site
Strategy-related challenges (S)	Customer requirements	Needs to improve understanding of customers’ markets to adjust accordingly and closely collaborate with customers. An increasingly individualised market requires a wider range of options, leading to the challenge of producing small order quantities				Challenged by global market with different demands and adjustments for different countries. “We need to manage to be “glocal”, both local and global. It is a nice word but it is very difficult to understand what it really implies.”
	Formalised operations strategy			Needs a common strategy, standardising the basic understanding of what the company is	Needs a clear strategic plan and to talk more about “what steps to take next”	
	Lean	Challenged by a “lack of strategy and how Kaizen activities link to such a strategy”. The strategic lean approach originates from corporate group, i.e., a top-down initiative with emphasise on fast set ups. Main challenge relates to communication: “we have not been able to communicate why this is important”		Associates the strategic aspects of lean with the concept of the “whole individual”, challenged to make lean the backbone of the organisation and to assure that the method is understood in the own organisational context		
	Long-term focus and strategic work	No outspoken operations strategy: “the strategy is to improve productivity by 10% and relates to the owners’ strategic philosophy”. “We do not have control over our processes, quality suffers due to too much rationalisations, being reactive rather than proactive”		Challenged by the organisational structure: “it is very difficult to work with strategies as there are so many levels”. Further challenged by “quarterly-capitalism” and its demands on return on investments within a year	Challenged by strategic work with lean throughout the organisation: “we have a rather uneven distribution of this knowledge”. Strategic investments are challenging as the return on investment requirement is quite short, two years, with great emphasis on the quarterly statements	Suffers from lack of strategic direction provided by the corporate group: “it is a challenge to understand how the market is changing and where our place will be”
	The role of owners	“This owner will never allow for large investments, have to make small investments and build on what we already have.” The owner lacks the feeling for being local, the site is “just a dot on the map, easily movable”			Challenged by the owner’s listing on the stock exchange and need for a steady cash flow: “there is no room for investments with long payback time”	Challenged by corporate group’s centralised decision processes on where to locate manufacturing activities, i.e., the intra-group competition. Concerned about the local site’s role in the group, “we are just one of all the other sites”. Further challenged by global investment stoppage
	Strategic alignment and strategic consensus		Needs to “get information out in organisation, wearing it down, so that people have truly understood”	Needs everyone on board: “they do not only need to understand what and how we do things, but also why we do it”	Needs “a standard for how to work with targets”	
Supply chain-related challenges (SC)	Distance to market and customers			Challenging to supply distant markets due to tendency to store too much inventory to handle long delivery lead times, causing inventory monitoring problems		Needs to be located with proximity of the customers’ production sites to avoid large fines for causing production stoppage. However, challenged by the size of the Swedish automotive industry, unclear what offshoring would lead to
	Flexibility, high mix/low volume	Needs proximity to suppliers, material needs to be near for rapid reactions to changed market conditions	Needs to find new ways to distribute small batches, potentially directly to the end customers	Needs a very flexible supply chain to meet different customers’ requirements		Needs suppliers that can manage flexible demands
	Intra-group relationships and ownership	Change in ownership challenges the supply chain structure. The local site has lost control over many functions and is competing for survival				Faces intra-group competition, a constant battle where the goal is to “win most of the available business”
	Price incoming goods/material		Needs strategic purchasing at an advantageous price since raw material represents 80 percent of the selling price			Needs suppliers to understand the necessity of value chain wide cost reductions, “we need to be much better at getting competitive prices”. Faces competitive market prices for some incoming material, like steel
	Quality incoming goods/material	Needs to support suppliers to fulfil the quality demands		Poor quality on incoming goods risks “triggering a lot of costs in our production”		Impossible to change from the only supplier available despite poor raw material quality for items of great importance for production
	Stronger supply chains, supplier relationships	Needs to consolidate supplier base and develop better system for active supplier relationship management		Needs to integrate customers into the supply chain, observing the “end-to-end” perspective. Further, needs an improved internal supply chain: “better to compete as a supply chain, rather than as independent companies”		Dependent on few suppliers with no alternative options. Received support from the customer when dealing with suppliers’ delivery problems. Supplier relationship development is unbeneficial: “we finance their business, but the return is too low”
Market-related challenges (M)	Increased competition			Challenged by Chinese competition, with highly trained, extremely driven and ambitious work force, “we have to shape it up if we shall stand a chance”		Challenged by globalisation: “it is possible to produce almost everything everywhere, while our cost level is consistently high”
	Marketing the own brand as local, Made in Sweden	Decreased differences among European producers but might still be differences in customers’ attitudes towards Chinese products. However, smaller differences than ten years ago			“It is currently an advantage to be local, to be Swedish, but newer generations might necessarily not care in the future, viewing themselves as international constantly being introduced to worldwide news”	
	Rapid changes, turbulent business environments				Needs to prepare for rapid changes and adaptations to the market: “when customers require news, we cannot spend years on product development”	Faces “shorter and shorter” time spans from quoting an offer to production initiation: “the concept creation and development stages are almost gone, barely have time to test before customers want products”
	Reduced volumes of sales, products with reduced interest from market	Faces market changes, moving towards a paperless society, leading to decreased demand for some of the company’s products, “have to steal market shares from competitors”			Faces changes to housing situations: “abandon old shared laundry rooms in apartment blocks in favour of household’s individual machines. We survive based on exchange of existing machines”	Faces reduced sales figures due to fluctuations in the market and customer projects’ life cycles
General environment-related challenges (E)	Educational system, attractiveness of industry jobs	Challenged by “the educational system, primarily on high school level” that “does not fulfil industry’s need for competent workforce”	Faces a younger generation “considering industry jobs to be dirty”. Challenging to “make people interested in process industry” Links attractiveness to geographical location: “very difficult to attract well educated and skilled employees to a small rural town.” Must “collaborate more with schools” to address need of “people with higher education”. National challenge: “redundant workforce no longer needed for simple tasks causes great matching problem in Swedish society.”	Challenged by expectations university graduates have on work tasks: “they seem to only be interested in office jobs, as if they have been promised something fancy. In reality they have to start at a lower level and prove they can perform”	Challenging to attract people to industry jobs: “worried the educational system cannot provide competence needed in the future.”	Identifies gap between educational levels: “cannot only have university educated engineers to run development, also need skilled workers to produce the products. Lacks high school engineers working with maintenance and as production technician.”
	Political decisions, rules and regulations		Challenged by European Union subsidies that support production in southern and eastern Europe, causing overcapacity on a European level and unfair market conditions. Further, Swedish social security system poses challenges due to extra costs and planning difficulties. Strong employment regulations are challenging when disallowing testing employees. Moreover, challenged by local energy market and its prices		Increasingly difficult to fulfil legal requirements, traceability, safety and European directives. Further, challenged by domestic public procurement regulations only considering procurement cost, not product life cycle cost	
